# Lakes as Source of Cholera Outbreaks, Democratic Republic of Congo

**DOI:** 10.3201/eid1405.071260

**Published:** 2008-05

**Authors:** Didier Bompangue, Patrick Giraudoux, Pascal Handschumacher, Martine Piarroux, Bertrand Sudre, Mosiana Ekwanzala, Ilunga Kebela, Renaud Piarroux

**Affiliations:** * Health Ministry, Kinshasa, Democratic Republic of Congo; † Université de Franche-Comté, Besançon, France; ‡ Institut de Recherche pour le Developpement, Strasbourg, France; § Organisation Mondiale de la Santé, Kinshasa, Democratic Republic of Congo

**Keywords:** Cholera, Vibrio cholerae, outbreaks, environment, Democratic Republic of the Congo, public health, dispatch

## Abstract

We studied the epidemiology of cholera in Katanga and Eastern Kasai, in the Democratic Republic of Congo, by compiling a database including all cases recorded from 2000 through 2005. Results show that lakes were the sources of outbreaks and demonstrate the inadequacy of the strategy used to combat cholera.

The association between *Vibrio cholerae* and aquatic environments has long been studied, but emphasis has been almost exclusively placed on coastal areas such as the Bay of Bengal, the point of origin of cholera. There, outbreaks are closely linked to estuarine areas, where environmental *V. cholerae* strains emerge and then spread in human communities during the monsoon season (*1*) by attaching themselves to surfaces provided by plants, algae, and zooplankton (*2*,*3*). Some recent studies have investigated environmental and climatic factors that may encourage the spread of cholera in African countries (*4*,*5*); these studies also focused on coastal areas. Except for 2 case–control studies performed in Burundi and Kenya (*6*,*7*), little is known about the epidemiology of cholera in inland areas of Africa. A recent article, based on the analysis of 632 reports of cholera outbreaks worldwide, has shown that 87.7% of cholera cases occurred in sub-Saharan Africa and that the highest concentration of outbreaks was in the eastern provinces of the Democratic Republic of Congo (DRC) (*8*). In this country, dozens of emergency programs have been implemented by humanitarian organizations, national health services, and international agencies; they have, however, failed to achieve long-term control of cholera epidemics. To search for environmental factors that could explain the recurrence of cholera outbreaks, we conducted an epidemiologic study in 2 inland provinces of the DRC severely hit by cholera.

## The Study

From 2002 through 2005, reports of cholera cases and deaths from cholera were collected weekly from each health district of Katanga (497,076 km^2^, 9,598,380 inhabitants) and Eastern Kasai (170,103 km^2^, 6,713,009 inhabitants) with the help of local and national staff of the DRC Ministry of Health. The definition of a case-patient was “any person 5 years of age or older in whom severe dehydration develops or who dies from acute watery diarrhea”; the age limit was lowered to 2 years for cases associated with confirmed cholera outbreaks, as recommended by the World Health Organization (WHO) (*9*). Each new outbreak was confirmed by culture and identification of *V. cholera* O1 from 5 to 10 stool samples.

**Figure 1:**
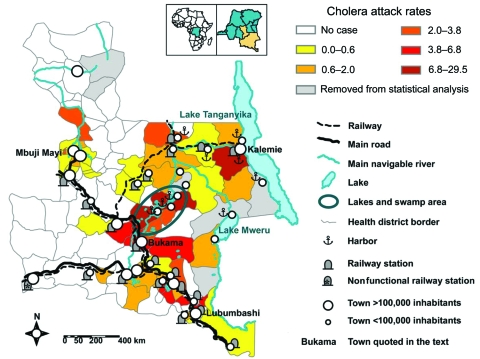
Katanga and Eastern Kasai, showing distribution of cholera attack rate from 2002 through 2005 and average attack rate of cholera per 10,000 inhabitants per health district.

For 2000 and 2001, only cumulative data collected weekly in each province were available; no detailed database was kept. However, data were completed with information from reports of epidemic investigations and interventions (105 reports filed from 1999 through 2005) and the testimonies of medical teams interviewed during field visits. A geographic information system was established, based on the data collected from the 106 health districts of the 2 provinces. Six health districts were removed from statistical analysis because >10% of weekly reports were missing (Figure 1). Using regression techniques (Technical Appendix), we statistically examined the relationship between the number of cholera cases in each health district and the following list of geographic and environmental variables: area; population; and presence/absence of cities of >100,000 inhabitants, of railway stations, of harbors, of major tracks or roads, and of lakes.

**Figure 2:**
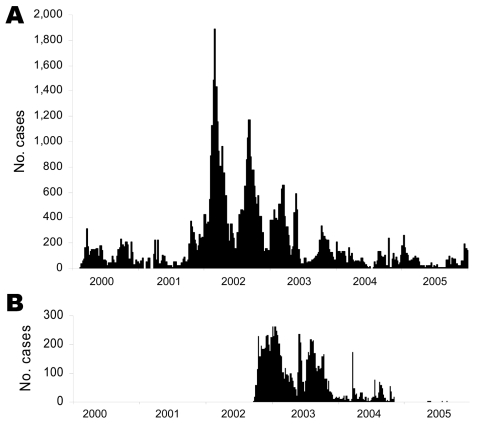
Weekly case incidence of cholera in Katanga (A) and Eastern Kasai (B) from 2000 through 2005.

A total of 67,738 cases and 3,666 deaths (case-fatality rate 5.4%) were reported from 2000 through 2005 in Katanga and Eastern Kasai, which corresponded to 8.4% of cases and 19.6% of deaths worldwide recorded by WHO during the same period (*10*–*15*). Relatively calm periods were separated by episodes of exacerbation between 2001 and 2003 (Figure 2). In 2000, epidemics were reported only in the areas of Lake Tanganyika and Lake Mweru (on the eastern border of Katanga). Only a brief outbreak (752 cases) was reported in Lubumbashi, the capital of Katanga, located in the south. The first exacerbation began in the middle of 2001, when thousands of civilians, trying to escape civil war. fled from Kalemie (bordering Lake Tanganyika). From May to December 2001, cholera outbreaks were reported in various cities of Katanga, including Bukama (center of Katanga) and Lubumbashi. From Bukama, outbreaks spread to the lakes north of the city. A peak was reached in March 2002, followed by a period of marked decrease, during which cholera persisted only in lake areas. A second exacerbation began in Bukama in August 2002, when fishermen returned from the lakes to sell their catches. This outbreak rapidly spread to Mbuji Mayi in Eastern Kasai where, as discovered during the outbreak investigation, the first case was in a tradeswoman who had traveled by train to Bukama to buy fish. In Mbuji Mayi, the cholera epidemic lasted until June 2004 and accounted for 4,949 cases. Concomitantly, in September 2002, another outbreak started in Lubumbashi; it lasted 9 months and affected 4,288 people.

Since July 2004, cholera has persisted only in lake areas. In the north of Bukama, this persistence has been due to iterative outbreaks, successively affecting cities and villages on the lakeshores. Cholera has also persisted on the shores of Lake Tanganyika, especially in the health district of Kalemie, where no lasting intermission has been recorded.

In our study, most cases (60%) occurred in lake areas (13% of inhabitants of the 2 provinces) (Figure 1). The number of cholera cases was statistically significantly higher in the presence of a lake, a main road, a harbor, or a railway (Table). The analysis of reports filed after each intervention showed that they were not conducted in the same way in each area. For every cholera outbreak in Mbuji Mayi, Lubumbashi, and other main cities in southern Katanga, medical staffs were reinforced by humanitarian organizations to set up centers for cholera treatment and to implement public awareness and information campaigns. In contrast, in lake areas, humanitarian organizations intervened in only 17 of 54 outbreaks. Interventions almost exclusively targeted patient care and sensitization campaigns were rarely implemented. Mean duration of interventions was 6 weeks (range 2–20) in lake areas versus 20 weeks in big cities (range 10–30).

**Table:** Model parameters and odds ratios of the negative binomial model selected for cholera cases in Katanga and Eastern Kasai, Democratic Republic of the Congo, 2000–2005*

Characteristic	Coefficient estimate	Standard error	t value	Pr(>|t|)	Odds ratio	95% CI
Intercept	5.50	1.04	5.27	8.92 × 10^−7^		
Ln (area)	−0.28	0.11	−2.44	1.64 × 10^−2^		
Population	1.97 × 10^−6^	1.17 × 10^−6^	1.69	9.46 × 10^−2^		
Railway station	0.61	0.25	2.42	1.74 × 10^−2^	1.8	1.12–3.02
Harbor	1.39	0.33	4.16	7.06 × 10^−5^	4.0	2.09–7.75
Main road	1.43	0.41	3.50	7.09 × 10^−4^	4.2	1.88–9.32
Lake	2.01	0.33	6.12	2.20 × 10^−8^	7.5	3.92–14.23

*Coefficient estimate, regression coefficients (for discrete variables, their exponential gives the odds ratio); t value, value of the t distribution; Pr(>|t|), probability of the null hypothesis of a coefficient estimate not statistically different from zero; CI, confidence interval; intercept, average number of cases; Ln, natural logarithm.

## Conclusions

Our study shows that, despite the difficulties encountered in gathering reliable field data in a country with a civil war and a disorganized healthcare infrastructure, a consistent set of results that help to understand the epidemiology of cholera in the DRC could be obtained. Information pooled from maps of the spatial distribution of cases, statistical analyses, and screening of investigation reports shows that lake areas are the source of iterative outbreaks and that these sometimes spread to main cities hundreds of kilometers away because of the movements of traders and other travelers. Although we fully acknowledge that the correlative nature of this study calls for further research to understand the details of the local natural history of cholera, our results show that the strategy used until now to combat cholera should be reexamined. Maximum effort must be concentrated on populations living around the lakes. Cholera prevention programs should be reinforced there, and safe water should be provided, especially during cholera outbreaks. Because the targets of the programs are relatively small populations living close to lakes, these programs could be more easily afforded than those implemented on a provincial or a national level. In this way, main cities, which are still under the threat of a new outbreak spreading from the lakes, would be protected.

## Supplementary Material

Technical Appendix

## References

[1] Colwell RR. Infectious disease and environment: cholera as a paradigm for waterborne disease. Int Microbiol. 2004;7:285–9. 15666250

[2] Reidl J, Klose KE. *Vibrio cholerae* and cholera: out of the water and into the host. FEMS Microbiol Rev. 2002;26:125–39. 10.1111/j.1574-6976.2002.tb00605.x 12069878

[3] Islam MS, Drasar BS, Sack RB. The aquatic flora and fauna as reservoirs of *Vibrio cholerae*: a review. J Diarrhoeal Dis Res. 1994;12:87–96. 7963350

[4] Mendelsohn J, Dawson T. Climate and cholera in KwaZulu-Natal, South Africa: the role of environmental factors and implications for epidemic preparedness. Int J Hygiene Environ Health; 2007 [cited 2008 Feb 18]. In press. Available from http://www.sciencedirect.com 10.1016/j.ijheh.2006.12.002 17383231

[5] Constantin de Magny G, Guegan JF, Petit M, Cazelles B. Regional-scale climate-variability synchrony of cholera epidemics in West Africa. BMC Infect Dis. 2007;7:20. 10.1186/1471-2334-7-20 17371602 PMC1839095

[6] Birmingham ME, Lee LA, Ndayimirije N, Nkurikiye S, Hersh BS, Wells JG, Epidemic cholera in Burundi: patterns of transmission in the Great Rift Valley Lake region. Lancet. 1997;349:981–5. 10.1016/S0140-6736(96)08478-4 9100624

[7] Shapiro RL, Otieno MR, Adcock PM, Phillips-Howard PA, Hawley WA, Kumar L, Transmission of epidemic control *Vibrio cholerae* O1 in rural western Kenya associated with drinking water from Lake Victoria: an environmental reservoir for cholera? Am J Trop Med Hyg. 1999;60:271–6. 10072150 10.4269/ajtmh.1999.60.271

[8] Griffith DC, Kelly-Hope LA, Miller MA. Review of reported cholera outbreaks worldwide, 1995–2005. Am J Trop Med Hyg. 2006;75:973–7. 17123999

[9] World Health Organization. Guidelines for cholera control. 2nd ed. Geneva: The Organization; 1996.

[10] World Health Organization. Cholera, 2000. Wkly Epidemiol Rec. 2001;76:233–40. 11505731

[11] World Health Organization. Cholera, 2001. Wkly Epidemiol Rec. 2002;77:257–64. 12189690

[12] World Health Organization. Cholera, 2002. Wkly Epidemiol Rec. 2003;78:269–76. 15581214

[13] World Health Organization. Cholera, 2003. Wkly Epidemiol Rec. 2004;79:281–8. 15315149

[14] World Health Organization. Cholera, 2004. Wkly Epidemiol Rec. 2005;80:261–8. 16106791

[15] World Health Organization. Cholera 2005. Wkly Epidemiol Rec. 2006;81:297–307. 16888873

