# Supplementary material for: Lakes as Source of Cholera Outbreaks, Democratic Republic of Congo
Source: Emerg Infect Dis. 2008 May;14(5):798–800. doi: 10.3201/eid1405.071260 (PMC2600234; doi:10.3201/eid1405.071260)
Supplement: Technical Appendix [file 07-1260_Techapp-s1.pdf]

Technical Appendix

Statistical Methods

The effect of geographical and environmental variables on the number of cholera cases in each health district was tested using a general linear model with a negative binomial distribution. Computing and graphs were done using R 2.4 (R Development Core Team, 2006), several R packages (maptools (16), sp (17), GAMLSS (18) and geoR (19)), and ArcGIS 8.3. Due to the overdispersion of cholera incidence, several kinds of generalized linear models were compared using quasi-Poisson, and type I and type II negative binomial distributions and they were checked for spatial structure. Stepwise selection of variables was performed in each case and the best models of each family were compared using the Akaike index criterion, according to Venables and Ripley (20) and Rigby et al. (21). The relationship between the number of cholera cases in health districts and geographical variables was finally modeled using the type II negative binomial family (log link function for both the mean and the distribution parameter). Distribution formula was as follows:

$$f(y\,|\mu,\sigma\,)=\frac{\Gamma(y+(\mu/\sigma))\sigma^{y}}{(\Gamma(\mu/\sigma)\Gamma(y+1)(1+\sigma)^{(y+(\mu/\sigma))})}$$

where μ is the mean, σ the dispersion parameter and $\sqrt{(1+\sigma)\mu}$ the standard deviation. The residuals were checked for spatial structure by plotting an empirical variogram where the distances were computed depending on the geographical coordinates of the centroid of each health district. A variogram envelope was then computed by performing 1000 permutations of the residual values on the spatial locations and the envelope limits were then compared to the variogram.

References

1. Lewin-Koh NJ, Bivand R. R:The maptools Package. Tools for reading and handling spatial objects. R package version 0.6-13. Available from http://cran.mirroring.de/src/contrib/Descriptions/maptools.html (accessed June 11, 2007).

2. Pebesma EJ, Bivand RS. Classes and methods for spatial data in R. R-news 2005;5:9–13. Available from http://cran.r-project.org/doc/Rnews/Rnews_2005-2.pdf

3. Stasinopoulos DM, Rigby B, Akantziliotou C. GAMLSS: generalized additive models for location, scale and shape. R package version 1.6-0. Available from http://www.r-project.org/useR-2006/Abstracts/Stasinopoulos+Rigby+Akantziliotou.pdf (accessed June 11, 2007).

4. Ribeiro Jr. PJ, Diggle PJ. GeoR: A package for geostatistical analysis. R-news 2001;1:15–18. Available from http://cran.r-project.org/doc/Rnews/Rnews_2001-2.pdf

5. Venables WN, Ripley BD. Modern Applied Statistics with S. 4 ed. New York: Springer, 2002.

6. Rigby RA, Stasinopoulos DM. Generalized additive models for location, scale and shape (with discussion). Applied Statistics 2005;54:507–54.
